# Using social and behavioral science to address achievement inequality

**DOI:** 10.1038/s41539-024-00259-1

**Published:** 2024-07-10

**Authors:** Eddie Brummelman, Nienke van Atteveldt, Sharon Wolf, Jellie Sierksma

**Affiliations:** 1 Research Institute of Child Development and Education, University of Amsterdam, Amsterdam, the Netherlands; 2 Department of Clinical, Neuro-, and Developmental Psychology & Institute LEARN!, Vrije Universiteit Amsterdam, Amsterdam, the Netherlands; 3 Graduate School of Education, University of Pennsylvania, Philadelphia, PA USA; 4 Department of Developmental Psychology, Utrecht University, Utrecht, the Netherlands

**Keywords:** Education, Psychology

## Abstract

Achievement inequality has been on the rise. Globally, students from disadvantaged backgrounds perform worse academically than their peers, even with equal ability. This represents a significant loss of potential and perpetuates inequality. We organized this interdisciplinary Special Collection to uncover experiences that contribute to achievement inequality, identify interventions that reduce it, and sketch a research agenda. We hope it inspires scholars committed to addressing social problems through basic research.

Achievement inequality is a defining challenge of our time. Around the world, students from disadvantaged backgrounds perform worse academically than their more advantaged peers, even if their ability is the same^1,2^. This represents a significant loss of potential and perpetuates inequality across generations^3^. The achievement gap emerges early in development and widens with age^4^. At age 15, the gap equals three full years of schooling^5,6^. This is a global problem, occurring across high-, middle-, and low-income countries^7^. Despite large-scale efforts to combat inequality, it has increased steadily over the past 60 years^8,9^.

We believe that the social and behavioral sciences are well positioned to address achievement inequality. They have theories and methods that are needed to better understand—and eventually address through intervention—the types of experiences that cause, maintain, or reinforce achievement inequality. Unfortunately, however, much social and behavioral research occurs in disciplinary silos, which prevents scholars from identifying and studying novel questions that cut across disciplines. We organized this Special Collection to bring together insights across the social and behavioral sciences in addressing achievement inequality. Here, we integrate the insights gained from this collection along three guiding questions: (1) What kinds of experiences contribute to achievement inequality? (2) How can interventions address these experiences to reduce achievement inequality? (3) How can scholars bring together their diverse theories and methods across disciplinary borders to spearhead the next generation of research on achievement inequality? We adopt a developmental perspective, focusing on children from preschool through primary and secondary school. This allows us to identify early sources of inequality and to evaluate developmentally tailored solutions. We hope this collection will serve as an inspiration for scholars who are committed to using basic social and behavioral science to address social problems.

## Experiences that contribute to achievement inequality

The first set of studies in our collection examines how children from low socioeconomic status (SES) backgrounds see themselves and how they are seen by others. These perceptions can have serious downstream consequences, from limiting participation in classroom discussions to undermining academic achievement.

In this collection, Hofer et al.^10^—a collaboration between psychologists and educational scientists—investigated children’s self-views as mechanisms of achievement inequality. Children from low-SES backgrounds are often exposed to denigrating messages about their academic abilities. For example, their teachers perceive them as less intelligent, believe their intelligence is fixed and cannot be developed, and disproportionally assign them to lower educational tracks—even when these children perform as well as their peers^1^. Consequently, children from low-SES backgrounds may develop more negative views of their academic abilities, which can stifle motivation and undermine achievement. Using data from the 2018 Programme for International Student Assessment (PISA), including 520,729 15-year-old students from 70 countries, Hofer et al. demonstrated that those from low-SES backgrounds indeed had more negative self-views: They had lower self-perceived ability, lower self-efficacy, more of a fixed mindset, and a reduced sense of belonging. These self-views, in turn, predicted poorer academic achievement. Self-views partially mediated the association between SES and achievement, thus perpetuating achievement inequality. By investigating this across 70 countries, the authors were able to demonstrate that the effects differed meaningfully between countries, depending on a country’s level of social mobility and income inequality.

Although Hofer et al. demonstrated socioeconomic disparities in children’s self-views, with downstream consequences for achievement, their research does not speak to the origins of these disparities. What experiences cause children from low-SES backgrounds to develop negative beliefs about their academic abilities? Some of these experiences might occur in classrooms. Classrooms embody an ideal of meritocracy: By providing all students with the same desk, the same teacher, and the same materials, they create an illusion of a level playing field, suggesting that any difference in achievement is due to effort or ability^2,11^. Yet, even in classrooms, children might be treated differently based on their SES. Three studies in our collection—Renoux et al.^12^, Schoneveld and Brummelman^13^, and Sierksma^14^—address this possibility. They demonstrate that children from low-SES backgrounds may face unequal treatment in the classroom, often at the hands of well-intentioned teachers or peers, with serious repercussions.

First, teachers may provide fewer opportunities to children from low-SES backgrounds to participate in whole-class discussions. For example, preschoolers from low-SES backgrounds are called on less by the teacher and speak less without being called on, regardless of their language ability^15^. Combining insights from social psychology and sociology, Renoux et al.^12^ conducted preregistered research showing that the reduced participation of preschoolers from low-SES backgrounds affects how they are seen by their peers. When preschoolers saw that a classmate participated less in class, they explained this primarily in terms of inherent factors—revealing a so-called *inherence bias*^16^. For example, they were more likely to think that the classmate is not smart (i.e., an intrinsic factor) than to think the classmate has reduced access to educational resources (i.e., an extrinsic factor). Over time, these inferences may turn attention away from extrinsic factors and perpetuate the idea that children from low-SES backgrounds are simply less competent than their peers.

Second, teachers may send seemingly well-intentioned but discouraging messages to children from low-SES backgrounds. Working at the intersection of developmental psychology and educational science, Schoneveld and Brummelman^13^ show that teachers may sometimes behave in ways that are superficially positive but undermine the perceived competence of children from low-SES backgrounds. A preregistered experiment showed that when a child from a low-SES (vs. high-SES) background succeeded in school, teachers delivered more inflated praise, such as “You did *incredibly* well!” Teachers probably gave more inflated praise because they inferred that the child from a low-SES background had to work harder to achieve their success. Another preregistered experiment showed that when children learned that another child received inflated praise (while an equally performing classmate did not), they perceived this child as more hardworking but less smart. Thus, teachers’ inflated praise may inadvertently reinforce the view of children from low-SES backgrounds as less smart.

Third, once children are stereotyped as being less smart, they may be treated by classmates in ways that perpetuate their presumed lack of smartness. Integrating theories from social and developmental psychology, Sierksma^14^ demonstrated that children might perpetuate competence-based inequality by offering disempowering help (i.e., help that does not offer recipients an opportunity to practice and improve their skills, such as providing the right answer rather than a hint). In two preregistered experiments, children were introduced to two peers whom they later overheard were either good or not so good at a task. Children then had an opportunity to help those peers. Children provided more *empowering help* (e.g., hints) to peers portrayed as competent and more *disempowering help* (e.g., right answers) to peers portrayed as incompetent. Thus, once stereotypes have been formed, interactions between children in a classroom can perpetuate them.

In addition to the unequal treatment of children from low-SES backgrounds within classrooms, there are regional and national policies that perpetuate achievement inequality. One such policy is ability tracking^17^, where children from lower SES backgrounds are assigned disproportionately to lower, vocational tracks, even when their academic achievement is on par^18^. In this collection, Bardach et al.^19^ linked research on ability tracking to insights from educational science and psychology. Specifically, they examined a mechanism through which ability tracking might perpetuate achievement inequality: *track-related stereotypes*—generalized beliefs about students belonging to a certain track. In their study, 3,880 secondary school students rated how they thought others perceive students attending their own track (e.g., to what extent others perceive these students as stupid, dumb, unmotivated, and lazy). Preregistered analyses confirmed the presence of such stereotypes, showing that students from lower, vocational tracks believed that others had relatively negative perceptions of students in their track, while students from higher, academic tracks believed that others had relatively positive perceptions of students in their track. Students from mixed tracks were in-between. However, changes in such stereotypes over time were not consistently related to changes in engagement, self-perceived ability, or academic achievement over time. That is, even when children became more aware of the negative stereotypes about their track, they did not suffer academically. One possible explanation is that stereotypes are most consequential when they are held by others (rather than the self), leading to biased treatment that perpetuates inequality, even if students are not aware of the biased treatment^1^.

In some cases, being the subject of negative stereotypes can compel students to work harder in school, even when this has long-term mental and physical health costs^20^. Black American youth face racial stereotyping, especially in the domain of mathematics, which can undermine their academic achievement^21^. Students become aware of these stereotypes in elementary school and begin endorsing them in secondary school^22^. Black students may develop strategies to cope with the stereotypes they face. Some students may distance themselves from their racial identity, whereas others may distance themselves from academics. In this collection, Wilson and Matthews^23^ combined insights from psychology and educational science to argue that one group of students has long been overlooked: those who resist pejorative racial ideologies by persistently pursuing academic success. A ninth-grade student remarked: “Like it’s not a bad thing being Black, it’s not. That’s one of the best things to be in life. Like it’s good, but you realize that you have to work ten times harder, because people is judging you off of what they think like a stereotype or a statistic…” (p. 1). In a sample of secondary school students, Wilson and Matthews measured students’ self-beliefs and identified a small group of Black American students showing a so-called *resistors* profile. These students viewed both their race and the domain of mathematics as important reflections of who they are, and they experienced a strong drive for school success as a way to resist discrimination, stigma, and stereotypes against their racial group.

After secondary school, many students from low-SES backgrounds enter vocational education. In this collection, Finkenauer et al.^24^—a team of interdisciplinary social scientists—discovered that vocational-education students who experience financial scarcity (e.g., who often worry about money) are at risk of dropout. Why are these students at risk? One explanation is that adolescents who experience financial scarcity lose trust in institutions. For example, they may see authority figures in school as dishonest, unreliable, and incompetent. Reduced institutional trust, in turn, may lead adolescents to disengage from school. Indeed, preregistered analyses demonstrated that reduced institutional trust partly explained why adolescents who experienced financial scarcity dropped out. This can have serious consequences, as students who drop out have worse economic prospects, including a higher likelihood of ending up in poverty^25^.

Collectively, the papers in this collection identify crucial experiences that reinforce achievement inequality. Children from low-SES backgrounds are treated differently by teachers and peers, irrespective of their actual abilities and achievements. For example, they receive fewer opportunities to participate in whole-class discussions and receive more superficially positive feedback and unsolicited disempowering help. Over time, such treatment can lead to them being perceived as less competent, feeling less competent, and losing trust in institutions, including educational institutions. This erosion of confidence can hinder academic achievement and increase the likelihood of dropout. While some children resist the negative stereotypes they encounter, the long-term consequences of this coping strategy can be detrimental.

## Interventions that reduce achievement inequality

As the first set of studies has uncovered experiences that contribute to achievement inequality, the second set focuses on intervention strategies—how to develop, design, and deliver interventions in ways that reduce achievement inequality. While some interventions target individual students, others target classrooms, schools, or even national policies.

Connecting insights across neuroscience and psychology, Harms and Garrett-Ruffin^26^ discuss in this collection how growing up in poverty contributes to cumulative risk exposure, resulting in chronic stress that impacts neurocognitive development. Over time, this may undermine academic achievement and perpetuate the intergenerational cycle of poverty. The authors call for two types of intervention strategies to lessen the harmful effects of poverty: (1) neuroscience-based educational practices that reduce the impact of poverty on educational achievement, such as by removing sources of stress from the curriculum; and (2) policies at the national, state, and district levels that eradicate poverty and address negative stereotypes about those living in poverty, including anti-racism policies.

Some scholars have suggested that *personalized learning*—embracing students’ individual differences in the classroom by personalizing their learning experiences, often with the help of technology—could help address achievement inequality. In this collection, Dumont and Ready^27^ reviewed literature from the learning sciences, philosophy, psychology, and sociology to identify the conditions under which personalized learning can be beneficial. They argue that personalized learning is unlikely to create *equality of outcomes*. It could, however, contribute to *adequacy*, “[equipping] all students with the basic competencies to participate in society as active members and to live meaningful lives” (p. 1).

Achievement inequality is a pressing issue worldwide, but its causes and consequences differ across regions. In sub-Saharan Africa, an increasing number of children enroll in primary school, but their learning outcomes remain low^28^. One barrier to learning is child labor. Worldwide, approximately 160 million children engage in child labor^29^. In this collection, Wolf and Lichand^30^ developed an intervention with the aim of reducing child labor and improving learning in Cote d’Ivoire, the largest producer of cocoa in the world, where child labor is common^31^. Building on insights from economics, applied developmental psychology, and educational science, they developed an SMS-based nudge intervention and tested it through a preregistered randomized controlled trial. The intervention delivered nudges via SMS and audio messages to parents, teachers, or both, with the aim of raising parental engagement and teacher motivation. The messages included reminders, encouragement, and activities. Parents received messages like: “School is a space for everyone, including families. You are invited to come to school next week. See you there!” Teachers received messages like: “Ask every child in your classroom to write on the board what they would like to learn. Then, ask them to choose another student to help them, by working in pairs.” Overall, the intervention did not reduce child labor and did not raise learning outcomes, regardless of whether the intervention targeted parents, teachers, or both. Among children who had started off with low learning levels, however, the parent-focused intervention raised learning outcomes and, seemingly paradoxically, also raised child labor. Specifically, the intervention raised children’s domestic work (e.g., cleaning the house), but not their cocoa farming activities. Together, these findings suggest that raising parents’ or teachers’ motivation—without alleviating the systemic barriers they face—might not be sufficient for generating broad improvements in children’s learning outcomes.

Most approaches to reducing achievement inequality target students, teachers, or school-based policies. Rarely, however, do they target the content of the curriculum or tests. In this collection, Muskens et al.^32^ asked: Can achievement inequality be reduced by making tests more relevant to the everyday lives of children from low-SES backgrounds? They adopted a hidden talents approach—rooted in insights from biology, psychology, and anthropology—to theorize that some social and cognitive abilities are enhanced, rather than impaired, through adversity^33^. Extending this notion, the authors hypothesized that children from low-SES backgrounds would perform better on mathematics items if these items reflected topics of real-world importance to them (e.g., money, food, and social relationships). They tested this preregistered hypothesis using cross-sectional data from Trends in International Mathematics and Science Studies (TIMSS), including data from 5501,165 4^th^ and 8^th^ grade students from 58 countries. Contrary to the authors’ hypothesis, children from low-SES backgrounds scored 18% *lower* on items of real-world importance. One possible explanation is that these items were so salient to them that their attention was diverted away from the task. This suggests that well-intentioned attempts to make questions more relevant to children’s everyday lives could, in some cases, backfire for children from low-SES backgrounds.

Another approach to reducing achievement inequality targets mindsets: beliefs about the malleability of intellectual abilities. A *growth mindset* is the belief that students can develop their abilities through effort and education, whereas a *fixed mindset* is the belief that students’ abilities are set in stone. In this collection, Hecht et al.^34^ brought together a large team of psychologists and educational scientists to review literature on how cultivating a growth mindset culture can reduce achievement inequality. They argue that mindsets do not just reside within teachers’ and students’ minds; they exist within a classroom, representing shared beliefs about what it means to be a learner in that classroom. Teachers can create growth-mindset cultures by conveying that all students can learn and improve, such as by expressing high expectations for all students, treating mistakes as opportunities for learning, allowing students to take ownership of their learning process while being a source of support, and providing feedback on the process rather than on ability^35^. This set of practices has been shown to reduce achievement inequality. An intervention that encouraged teachers to adopt growth-mindset practices improved students’ achievement, especially in classes with a higher proportion of students from low-SES backgrounds^36^.

Together, the papers in this collection demonstrate the promises and perils of psychological interventions for reducing achievement inequality. Most existing psychological interventions target students individually, which could convey to students that the onus of change is on them^37,38^. Challenging this approach, several interventions in this collection target those in positions of power, such as teachers or policy makers, which can create fertile soil for psychological change in students. Sustainable change requires both “good seeds” (a psychologically wise intervention) and “fertile soil” (a context that affords the way of thinking offered by the intervention)^39^. For example, teaching children a growth mindset is unlikely to improve their academic achievement if their teachers continue to endorse a fixed mindset^40^. Thus, achievement inequality cannot be solved through individual psychological intervention alone; psychological interventions must be supplemented by structural changes—at the classroom, school, and national policy level—that create a fertile soil that enables students to put their new ways of thinking into practice.

## The next generation of research on achievement inequality

Looking into the future, what types of research do we need to advance our understanding of achievement inequality? Based on the promising examples showcased in our collection, we outline a roadmap for future research.

First, we call for research that challenges *deficit perspectives* on achievement inequality. According to these perspectives, children from low-SES backgrounds have deficits in their individual attributes—such as their intelligence, executive functions, self-regulation, or motivation—that lead them to underperform in school^41^. Such deficit perspectives may be fueled by society’s firm belief in meritocracy, which assumes that students’ achievements in school are reflections of their efforts and abilities. In our collection, Zengilowski et al.^42^ formed a unique team of educational, developmental, and cognitive psychologists to challenge deficit perspectives. They argue convincingly that overemphasizing individual attributes may lead one to overlook the actual structural causes of achievement inequality and may inspire individual interventions that put an increased burden on children from low-SES backgrounds. Thus, over time, a deficit perspective may both justify and perpetuate existing inequalities in education.

Several studies in our collection challenge the deficit perspective. One line of work, described by Muskens et al.^32^, is based on the premise that children from low-SES backgrounds develop skills that enable them to contend with the challenges of growing up in low-SES environments (e.g., detecting threats, finding creative solutions in times of scarcity, reading others’ intentions). That is, growing up in low-SES backgrounds does not invariably lead to deficits; it leads to a specialized set of skills that is often underappreciated in academic contexts. Another line of work, exemplified by Renoux et al.^12^ and Schoneveld and Brummelman^13^, shows that teachers treat children from low-SES backgrounds unfairly, even when their abilities and achievements are on par. Why? One explanation is that teachers perceive a mismatch between the tendencies of children from low-SES backgrounds and those that are valued in academic contexts^43^. For example, children from low-SES backgrounds may prefer working together, eschewing competition, being modest, and deferring to adults and peers, while academic contexts value working individually, embracing competition, being assertive, and challenging others. In other words, the attributes of children from low-SES backgrounds are not inferior to those valued in academic contexts—“they just happen to be *different*, and this difference should not be mistaken for a deficit”^43^. Yet, teachers may explain this mismatch in terms of deficits (e.g., perceiving children from low-SES backgrounds as less smart). Such inherent explanations may lead teachers to treat children from low-SES backgrounds in ways that exacerbate existing inequalities (e.g., excluding them from whole-class discussions and lavishing them with inflated praise for average achievements).

Together, these two lines of work suggest that solutions to achievement inequality should not attempt to fix the apparent deficiencies of children from low-SES backgrounds; rather, they should create environments that allow all children, regardless of their socioeconomic backgrounds, to fulfill their potential. Consistent with this approach, Hecht et al.^34^ show how creating a growth mindset classroom culture can reduce achievement inequality.

Second, we call for research at the intersection of disciplines. Achievement inequality is a multifaceted problem with many underlying causes. Only by working across the borders of disciplines can we fully comprehend and address these causes. In this collection, educational scientists Elffers et al.^44^ argue that, when forming such interdisciplinary collaborations, it is critical to establish a shared understanding by considering one’s implicit—and often discipline-specific—theories of achievement inequality. Are all forms of achievement inequality problematic, or are some forms acceptable or even desirable? Should teachers strive to provide children with equal opportunities, even if this results in unequal outcomes, or should they provide some children with more opportunities than they do others? Can educational institutions eventually reduce achievement inequality, or are they social sorting machines that are designed to maintain or even exacerbate existing inequalities? Asking these questions will help researchers align and sharpen their theories and methods, and, importantly, inform when and for whom interventions are most needed.

By collaborating across disciplinary borders, researchers can develop shared research infrastructure that will allow them to conduct the same study or test the same intervention in different contexts. This is critical for understanding *heterogeneity*: what works for whom under what conditions^45^. Without such a shared infrastructure, researchers are limited to meta-analyses that integrate studies with different designs, different measures, and different sampling strategies after they have been conducted. With a shared infrastructure, researchers can coordinate designs, measures, and sampling strategies in advance. And by pooling resources, they can purposefully recruit samples that are heterogeneous and generalizable. For example, rather than limiting themselves to children from high-SES backgrounds, they can target children from a wide range of SES backgrounds; or rather than limiting themselves to high-income countries, they can target low-, middle-, and high-income countries. Several papers in our collection set an example. Hecht et al.^34^ discuss the importance of large heterogeneous samples and multi-site studies for identifying the conditions under which growth-mindset interventions work. Hofer et al.^10^ used the PISA 2018 data to examine antecedents and consequences of socioeconomic disparities in self-views across 70 countries, allowing them to identify differences between countries. Lichand and Wolf^30^ tested their intervention in 100 schools in Cote d’Ivoire, including a total of 2246 students, which allowed them to compare intervention effects across child- and family-level characteristics. To create the necessary infrastructure, they established a collaboration with Movva, a Brazilian social enterprise, and partnered with the Ministry of Education and local schools. We hope to see more international multi-site research that explores the best contextualized solutions to achievement inequality globally.

Third, we call for research that develops and implements inclusive research methods. In this collection, a group of neuroscientists, educational scientists, and psychologists, Adams et al.^46^, describe a case in point. Over the past decades, findings from neuroscience have been used increasingly to inform theory and policy in the field of education. Electroencephalography (EEG) is an important tool in this field, given its high temporal resolution, its ability to be used outside of traditional lab settings, and its suitability for use with young children, even infants^47^. However, it is difficult to collect high-quality EEG data from participants with thick, voluminous, and curly hair types and certain hairstyles. Adams et al. describe how this has led to unintended systemic racism, as certain minority populations—such as Black and Latinx students—have been excluded disproportionally from EEG research. The authors provide a substantial list of recommendations for conducting EEG research with these populations, thereby making EEG research more inclusive.

How can researchers make sure that their research methods are inclusive? They can do so by engaging with and learning from the populations they study. They can involve the populations they study in every step of their research—from conceptualizing the research questions and developing the materials to interpreting the findings and identifying new research directions. This approach turns the populations into co-constructors of knowledge rather than mere objects of study, helping researchers avoid the deficit perspectives and exclusionary research methods that are still dominant in many fields^48^. Of course, the populations need to be properly compensated and credited for these efforts. Unfortunately, such an inclusive approach is often discouraged, because it is believed to interfere with objectivity and can be mistaken for bias, especially if the researcher is part of the population they study^49^. We hope that scholars can correct this misunderstanding by demonstrating that research can be both inclusive and rigorous.

## Concluding remarks

What kinds of experiences contribute to achievement inequality? And how can we address these experiences through intervention? Our special collection has attempted to address these questions by bringing together insights from different fields, including developmental psychology, social psychology, educational science, neuroscience, sociology, economics, and interdisciplinary social sciences. Each of these fields has a history of studying important social problems, and each field brings unique tools, from rich theories to rigorous methods. Yet, incentive structures in academia have discouraged some of the high-risk high-gain research that is needed to answer big questions. Our collection demonstrates that a commitment to social problems can reinvigorate our work^50^. It connects researchers across disciplinary boundaries, which enables them to look beyond their own theoretical borders, to engage in transformative interdisciplinary projects, and to build a shared research infrastructure. We hope this collection serves as an inspiration for a new era of research that addresses interdisciplinary, big, and bold questions.

### Reporting summary

Further information on research design is available in the Nature Research Reporting Summary linked to this article.

### Supplementary information

Reporting Summary
